# Behavioral Changes Without Respiratory Symptoms as a Presenting Sign of COVID-19 Encephalitis

**DOI:** 10.7759/cureus.10469

**Published:** 2020-09-15

**Authors:** Tania Rebeiz, Krista Lim-Hing, Shahab Khazanehdari, Karim Rebeiz

**Affiliations:** 1 Department of Neurosurgery, North Shore University Hospital, Manhasset, USA; 2 Department of Radiology, Memorial Sloan Kettering Cancer Center, New York, USA

**Keywords:** covid-19, encephalitis, encephalopathy, neurologic, central nervous system

## Abstract

The clinical presentation, diagnosis, and treatment of coronavirus disease 2019 (COVID-19) encephalitis are still being characterized. Few case reports describing COVID-19 encephalitis are available in the literature. We present a case of COVID-19 encephalitis who presented with behavioral disturbances without respiratory symptoms.

## Introduction

On March 11, 2020, the World Health Organization (WHO) officially announced the COVID-19 outbreak a pandemic [1]. To date, there have been approximately more than 24,299,923 cases resulting in over 827,730 deaths worldwide [2]. Although respiratory manifestation is the most common presenting sign, COVID-19 can also target the central nervous system. The projected prevalence of central nervous system involvement due to COVID-19 is a total of 1805-9671 cases [3]. Some of the neurologic manifestations reported include stroke, acute necrotizing encephalopathy, seizures, acute disseminated encephalomyelitis, and meningoencephalitis [4]. 

There are few case reports of COVID-19 encephalitis. However, these reports are limited by lack of cerebrospinal fluid (CSF) analysis, abnormal neuroimaging findings, and exclusion of other etiologies [5,6]. Moreover, most cases present in the setting of pulmonary involvement, with only two cases of isolated COVID-19 encephalitis described in the literature [1,5]. Here, we report a case of COVID-19 encephalitis presenting with behavioral disturbances without respiratory symptoms.

## Case presentation

**Figure 1:**
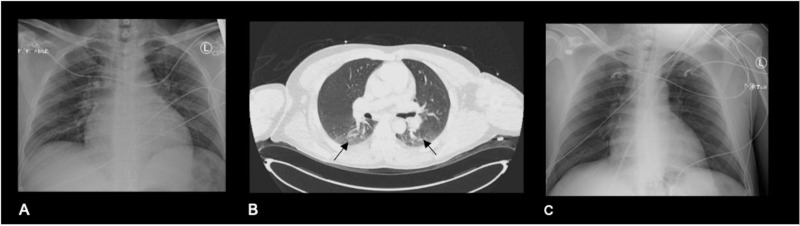
Chest imaging (A) Posteroanterior (PA) chest X-ray on admission demonstrates clear lung. (B) Axial CT chest demonstrates clear lungs with slight atelectasis in the dependent portion of the lungs (black arrows). (C) Chest X-ray on readmission was still clear.

A previously healthy man in his early thirties was brought to the emergency department for the evaluation of behavioral changes, which according to his friends was described as “not acting like himself.” The patient had prior history of alcohol abuse and his last drink was more than 10 days prior to presentation. On physical examination, he was comfortably breathing on room air but was febrile, confused, and intermittently following commands without evidence of focal neurological deficits. He had negative Kernig’s and Brudzinski’s signs. Given the presence of fever and the ongoing COVID-19 pandemic, real-time reverse transcription polymerase chain reaction (rRT-PCR) testing was performed and was positive for severe acute respiratory syndrome coronavirus 2 (SARS-CoV-2) RNA. Chest X-ray and CT chest were clear (Figure 1A, 1B).

**Figure 2:**
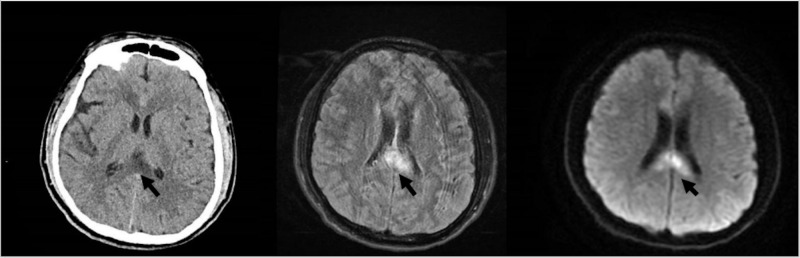
Brain imaging on first admission (A) Axial CT brain image demonstrates a nonspecific hypoattenuation in the splenium of the corpus callosum (black arrow) and questionable subarachnoid hemorrhage (not shown). (B and C) Axial MRI brain FLAIR and diffusion images demonstrate abnormal FLAIR hyperintense and diffusion restricting lesions at the splenium of the corpus callosum correlating with the CT findings (black arrows). MRI did not show any subarachnoid hemorrhage. FLAIR, fluid-attenuated inversion recovery

Urine toxicology was negative. Cranial CT on admission revealed no acute abnormalities except for a questionable subarachnoid hemorrhage within the mesial parietal region and nonspecific hypoattenuation in the splenium of the corpus callosum (Figure 2A). This lead to MRI and magnetic resonance venography, which were only notable for diffusion restriction and T2/fluid-attenuated inversion recovery (FLAIR) hyperintensity involving the splenium of the corpus callosum (Figure 2B, 2C). CT angiography (CTA) was unremarkable. The patient was hospitalized for approximately three weeks. His stay was notable for psychotic features including hallucinations which prompted psychiatric consultation and the prescription of antipsychotics. He was supplemented with thiamine and vitamin B12. His mental status improved, and he was discharged with the presumed diagnosis of Marchiafava-Bignami disease. 

**Figure 3:**
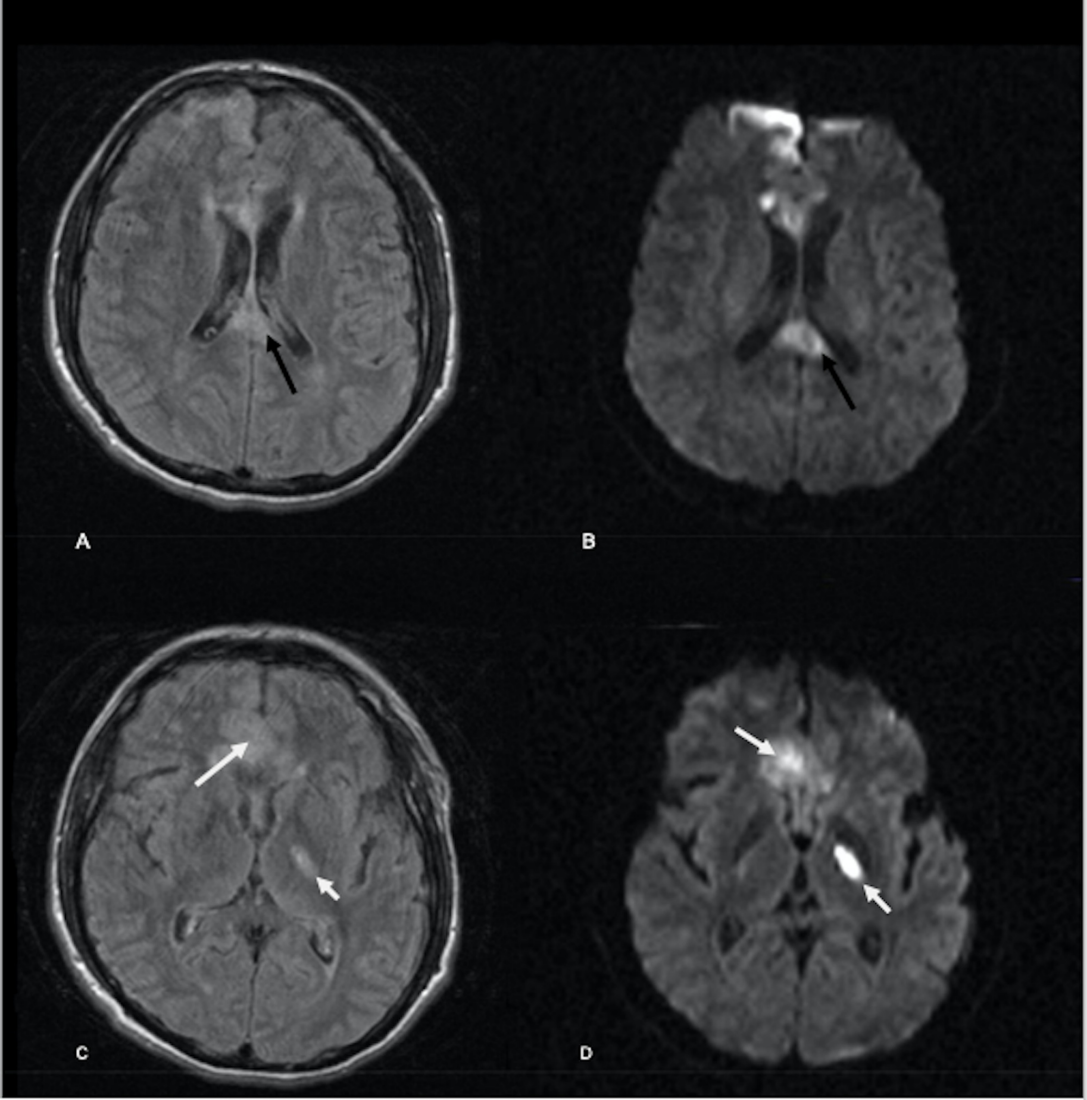
Brain MRI on second admission Axial MRI brain FLAIR and diffusion images on readmission. (A-D) Axial FLAIR and diffusion images demonstrate a slight decrease in the previously visualized restricting corpus callosum lesion (black arrows). However, new FLAIR hyperintense and diffusion restricting lesions are seen in the right para sagittal frontal cortex and left thalamus (white arrows). FLAIR, fluid-attenuated inversion recovery

The patient was readmitted five days later due to worsened mental status. On examination, he was nonverbal, awake, and with no focal neurological deficits. A follow-up rRT-PCR testing was negative for SARS-CoV-2 RNA. Chest X-ray was clear (Figure 1C). Brain MRI was remarkable for new abnormal T2/FLAIR hyperintense and restricted diffusion involving the left thalamus, right parasagittal frontal cortex, and bilateral genu of the corpus callosum (Figure 3). No leptomeningeal or parenchymal enhancement was seen. The patient was started on empiric acyclovir, ceftriaxone, and vancomycin for presumed meningoencephalitis. CSF analysis revealed lymphocytic pleocytosis with CSF-white blood cell (WBC) of 350 cells/µl of which 98% were mononuclear cells. Moreover, CSF protein was 297 mg/dl and glucose was 56 mg/dl. CSF culture was negative. CSF PCR assays for herpes simplex virus 1 (HSV-1), herpes simplex virus 2 (HSV-2), varicella zoster virus (VZV), cytomegalovirus (CMV), human herpes virus 6 (HHV-6), and enterovirus were negative. Repeat inflammatory markers including C-reactive protein (CRP) and interleukin-6 (IL-6) were within normal range. Erythrocyte sedimentation rate (ESR) was 27 mm/hr. Electroencephalography revealed generalized slowing but no epileptiform discharges. 

**Figure 4:**
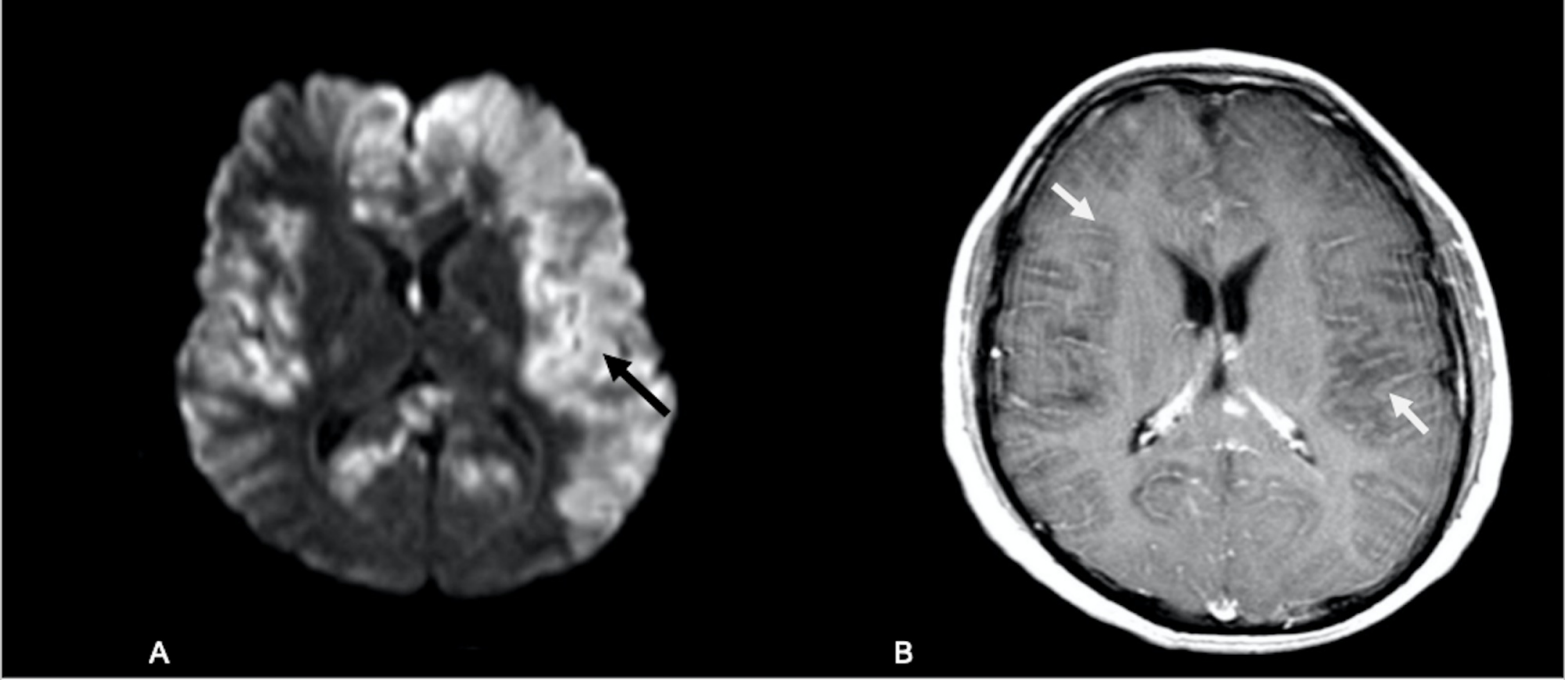
Follow-up brain MRI (A) MRI brain diffusion images demonstrate a marked increase in extent of left greater than right hemispheric diffusion restriction (black arrows). (B) Axial T1 postcontrast images demonstrate new widespread sulcal enhancement (white arrows).

Due to neurological deterioration, the brain MRI was repeated a week later and demonstrated marked progression of the restricted diffusion now involving the bilateral frontal, parietal temporal and occipital lobes, corpus callosum and basal ganglia (Figure 4A), and new sulcal enhancement throughout the cerebral hemispheres (Figure 4B). The patient’s neurological examination rapidly worsened; he became obtunded and developed a left gaze preference and right-sided weakness. He started having seizures controlled with antiepileptics. Due to the continued clinical deterioration, he was transferred to a tertiary care center, where he required intubation due to acute respiratory failure. He continued to deteriorate, with worsening brain edema. Despite hyperosmolar therapy, he progressed to brain death on day 49 from first symptom onset. 

## Discussion

Encephalitis is an inflammation of the brain that can be infectious or immunologic in etiology. A total of 13 cases of COVID-19 meningoencephalitis have been reported in the literature [5]. The postulated mechanisms are direct invasion of the central nervous system and secondary immune-mediated inflammatory response to the virus [4]. Some of the presenting signs are altered mental status, fever, seizures, and neck stiffness. There is only one case report of COVID-19 encephalitis presenting with psychotic features [7]. Although most present with respiratory symptoms days before the neurological manifestations, there are only two reported cases of isolated COVID encephalitis [3]. Our patient presented with psychotic features without respiratory symptoms, with clear lungs on chest X-ray and unremarkable CT chest. 

Diagnostic tests include neuroimaging, CSF analysis, and electroencephalography. Our patient’s first MRI finding revealed FLAIR hyperintensity in the splenium of the corpus callosum. His follow-up MRI brain showed evolution in the FLAIR hyperintensities and diffusion restricting lesions in the cortical and deep gray matter that progressively worsened over days. A recent study evaluating the phenotype of COVID-19 encephalitis found that almost half of the patients had normal MRI findings; however, some of the patients with abnormal MRI findings had subcortical and cortical T2 and diffusion-weighted imagining (DWI) hyperintensities [8]. In addition, all of the patients had abnormal EEG readings which included generalized slowing and epileptiform discharges. Of the 13 reported cases of COVID-19 encephalitis, only 4 had CSF lymphocytosis and 2 tested positive for the SARS-CoV-2 RT-PCR [3]. A recently published review article classifies the diagnosis of SARS-CoV-2 encephalitis/meningitis as confirmed, probable, or possible. The diagnosis is confirmed if there is detection of SARS-CoV-2 in the CSF or brain, or the presence of antibodies in the CSF, with lack of other explanatory causes. If the antibodies or the SARS-CoV-2 is detected only in the respiratory sample, with lack of other possible etiologies, the diagnosis of COVID-19 encephalitis is probable. Our patient had CSF lymphocytic pleocytosis with CSF analysis which was unremarkable for other infectious etiologies. His MRI findings were suggestive of encephalitis, and his EEG revealed generalized slowing. Unfortunately, due to the limited testing during the pandemic, our patient’s CSF was not analyzed with SARS-CoV-2 RT-PCR.

Treatment is still unknown; however, there are case reports showing good response to immunosuppressive therapies [9]. Due to the delay in the diagnosis and rapid neurological decompensation, our patient was not treated with immunosuppressive therapies. We recommend considering COVID-19 encephalitis in patients presenting with isolated behavioral disturbance during the COVID pandemic.

## Conclusions

This case highlights that COVID-19 encephalitis can present with isolated behavioral disturbances without respiratory symptoms. If left untreated, COVID-19 encephalitis can lead to devastating brain injury and brain death. 
